# Healthcare professionals’ beliefs on promoting physical activity in oncology: a COM-B framework analysis

**DOI:** 10.1007/s00520-026-10635-9

**Published:** 2026-04-15

**Authors:** Eliana V. Carraça, Ana Leonor Oliveira, Gonçalo Carvalho, Mariana Clemente, Tiago Resende, Catarina Ribeiro, António Palmeira, Bruno Rodrigues

**Affiliations:** 1 CIDEFES, Faculdade de Educação Física e Desporto, Universidade Lusófona & CIFI2D, Faculdade de Desporto, Universidade do Porto, Campo Grande 376, 1749-024 Lisbon, Portugal; 2 CIBB, Faculdade de Medicina, Universidade de Coimbra, R. Larga 2, 3000-370 Coimbra, Portugal

**Keywords:** Beliefs, Physical activity, Exercise, Cancer, Healthcare professionals

## Abstract

**Purpose:**

This study was aimed at exploring the opinions and promotion practices of physical activity and structured exercise among healthcare professionals—both physicians and non-physicians—in oncology care and at analyzing the associations between their beliefs/attitudes, perceived capability, opportunity, and motivation and their promotion practices.

Methods

This cross-sectional study included 152 physicians (71.1% women; 38.9 ± 10.5 years) and 59 non-physician health professionals (78.9% women; 42.2 ± 8.7 years) working in Portugal, who completed an online survey between 2019 and 2024. Data were analyzed using Mann–Whitney tests for dichotomous outcomes, Spearman’s correlations for continuous variables, and multiple regression analyses.

**Results:**

Although most professionals acknowledged the importance of physical activity and reported positive beliefs, many felt unprepared to prescribe structured exercise and did so infrequently. Among physicians, the strongest predictors of promotion practices were enjoyment in discussing exercise with patients (reflecting intrinsic motivation), perceived opportunity (having time during consultations), and perceived competence to prescribe structured exercise. For non-physician professionals, perceived knowledge and competence, enjoyment in discussing physical activity, and valuing its health benefits were most relevant.

**Conclusion:**

These findings highlight the need to enhance training in physical activity and exercise prescription among oncology healthcare providers to strengthen knowledge and competence and improve promotion practices. Future research should confirm these findings using larger samples and longitudinal designs.

## Introduction

Physical activity (PA) is now widely recognized as a key component in the prevention, treatment, and rehabilitation of cancer [1]. Its benefits are well documented and include reductions in treatment-related side effects such as fatigue, improvements in physical functioning and quality of life, and even lower risks of recurrence, comorbidity, and mortality [2, 3]. Nonetheless, engagement in PA remains low among cancer survivors, with approximately 36.7% of cancer survivors being physically inactive during their leisure time and only 15.9% meeting the recommended guidelines for both aerobic and muscle-strengthening activities [4]. 

One factor that may help increase PA participation among cancer survivors is professional guidance from healthcare providers. Studies consistently show that survivors are more likely to adopt and maintain physically active lifestyles when PA is recommended by a health professional, particularly an oncologist [5–7]. Patients tend to perceive oncologists as credible and trustworthy sources of health information, which places these professionals in a strategic position to encourage health-promoting behaviors, including PA. However, evidence suggests that few oncologists actively promote PA or refer their patients to qualified exercise professionals [6, 8–10].

Understanding why these missed opportunities occur has become an important line of research. Several studies have identified barriers that prevent oncologists from routinely addressing PA, including limited time during consultations, lack of knowledge about exercise prescription, safety concerns, and an absence of institutional support or referral pathways [9, 11, 12]. In a previous study, both patients and professionals not only highlighted the importance of oncologists in promoting PA but also revealed multiple barriers, including unclear professional roles, fragmented care pathways, and insufficient training and confidence among clinicians [13]. Notably, professionals reported that even when they believed PA was beneficial, structural and interpersonal barriers frequently undermined their capacity to act on these beliefs.

These observations align with the COM-B model [14], a behavioral framework that posits that Capability, Opportunity, and Motivation are essential prerequisites for the enactment of any behavior. Within this framework, *capability* refers to the individual’s physical and psychological ability to perform the behavior, including skills, knowledge, and confidence. *Opportunity* includes external factors such as environmental resources, social norms, and institutional infrastructure that make the behavior possible or prompt it. *Motivation* encompasses the cognitive and emotional processes that direct behavior, both reflective (e.g., beliefs about outcomes) and automatic (e.g., habits and emotional responses).

In the oncology context, studies suggest that clinicians often lack the psychological capability to promote PA, due to insufficient training in exercise prescription or uncertainty about how to tailor recommendations to different cancer types, stages, or treatment phases [9, 11]. At the level of opportunity, many professionals report a lack of time during consultations, insufficient access to referral networks, and few institutional supports to facilitate PA promotion [10, 12]. Motivationally, while most oncologists acknowledge the benefits of PA, some remain concerned about the safety of exercise for certain patients or feel ambivalent about their role in promoting lifestyle changes [11, 12].

Attitudes and behavioral beliefs play a central role in this equation. According to the Theory of Planned Behavior [15], individuals form intentions to act based on their attitudes (positive or negative evaluations of a behavior), subjective norms (perceived social pressure), and perceived behavioral control (confidence in their ability to perform the behavior). Beliefs about outcomes—such as whether PA will genuinely benefit patients or whether it is safe during chemotherapy—strongly influence oncologists’ attitudes and thus their intentions to counsel on PA. If clinicians hold favorable beliefs and feel competent and supported, they are more likely to engage in PA promotion.

In Portugal, similar trends have been observed. Rodrigues et al. [12] found that many oncologists felt unprepared to advise on PA due to a lack of training and time, which limited their actual promotion behaviors. However, these professionals acknowledged the importance of exercise physiologists and saw collaboration with such specialists as essential. Likewise, Sequeira et al. [16] found that survivors of breast cancer were more likely to receive appropriate PA recommendations when their healthcare providers had positive attitudes toward PA and understood its benefits. Conversely, providers with safety concerns or limited confidence tended to avoid discussing PA altogether.

Taken together, these findings highlight the relevance of exploring and understanding health professionals’ beliefs, attitudes, capability, opportunity, and motivation, in order to design effective strategies to support PA promotion in oncology settings. Given this context, the present study is aimed at exploring the views and practices of oncology healthcare professionals in relation to the promotion of PA and exercise among cancer patients. Specifically, we sought to understand whether professionals’ beliefs and attitudes toward PA, along with their perceived capability, opportunity, and motivation to promote it, were associated with their actual counseling behaviors. Grounded in the COM-B model [14] and informed by previous research [11–13], we hypothesized that professionals with lower perceived competence or knowledge, limited access to supportive environments or referral structures, and weaker motivation to engage in PA counseling would report fewer promotion practices. Conversely, we expected that those who held more favorable beliefs and attitudes toward PA—believing it to be beneficial, safe, and within their professional remit—would be more likely to recommend or encourage PA among their patients.

By investigating these relationships, this study is aimed at identifying the key psychological and contextual drivers of PA promotion in oncology care. Such insights may inform future strategies to support healthcare professionals in embedding PA into routine clinical practice and ensure that more cancer patients benefit from its well-documented advantages.

## Methods

### Study design

This was a cross-sectional observational study. An online survey on PA promotion beliefs, perceptions, and practices among health professionals working in oncology was disseminated through a Google Forms’ link, between 2019 and 2024, with the help of medical oncologists, mailing lists, social media, academic networks, and oncology associations. Participants signed an informed consent form before completing the survey. All procedures were approved by the relevant institutional ethics board and complied with the Declaration of Helsinki.

### Participants

A total of 211 professionals participated: 152 physicians (71.1% female; mean age = 38.9 ± 10.5 years) and 59 non-physicians (78.9% female; mean age = 42.2 ± 8.7 years). Most physicians worked in non-university hospitals (47.3%) and had an average of 6.1 ± 6.7 years of oncology experience. Only 21.7% had received any PA-related training. Non-physicians were mostly nurses (79.3%), with an average of 17.4 ± 9.2 years of experience; 79.3% had no formal training in PA or exercise.

### Measures

A structured online survey, based on Rodrigues et al. [12] and other international studies [5, 6, 8–10] was developed using the COM-B framework [17]. It assessed the following: (1) Capability (4 items; e.g., self-reported knowledge/competence to prescribe exercise); (2) Opportunity (2 items; e.g., time and resources available); (3) Motivation (1 item); (4) Beliefs and attitudes (5 items; e.g., perceived importance of PA for patients); and (5) Promotion practices (5–6 items), adapted to clinical roles. Beliefs and attitudes toward PA were captured with five additional items (e.g., “Raising awareness of structured exercise is important for improving patient health”). All responses used five-point Likert scales (“strongly disagree” to “strongly agree”). Items were analyzed individually rather than combined into composite scores. Demographic questions covered age, sex, years in oncology (or general practice for non-physicians), job grade, hospital type, consultation length, and prior PA training.

### Statistical analysis

Data were analyzed with JASP 0.19.2.0.

Descriptive statistics summarized professionals’ opinions and promotion practices, using means ± standard deviations for continuous variables and absolute and relative frequencies for categorical ones. Normality was checked with the Shapiro–Wilk test, revealing non-normal distributions. Spearman correlations were conducted to test associations between COM-B constructs (capability, opportunity, and motivation) or beliefs/attitudes and PA promotion behaviors, measured as ordinal outcomes. For dichotomous outcomes (“Do you promote PA?”/“Do you promote structured exercise?”), Mann–Whitney *U* tests were conducted to assess differences between Yes/No outcome levels in COM-B constructs and beliefs/attitudes. To identify the most salient predictors of promotion behavior, we performed exploratory multiple regression analyses—linear for ordinal outcomes and logistic for dichotomous ones—using the stepwise method. Statistical significance was set at *p* = 0.05.

## Results

Regarding the promotion of PA, 94.7% of physicians and 87.9% of other health professionals reported encouraging PA among their patients. However, only 44.4% and 54.9% reported recommending structured exercise, respectively. Physicians had an average consultation time of 25.8 ± 9.5 min, with 3.8 ± 3.0 min dedicated to promoting PA. Structured PA was prescribed in 52.7 ± 27.3% of consultations. Non-physician professionals recommended PA to 64.8 ± 29.3% of their patients and structured exercise to 35.6 ± 31.2%. When asked about referring patients to exercise physiologists, nearly all participants stated they would do so if such professionals were integrated into the healthcare system (98% of physicians and 100% of other health professionals). However, at the time, their habit of referring patients to exercise professionals was very low (mean = 1.5 ± 0.9 on a 1 to 5 scale).

To analyze the associations between relevant variables, comparisons were made between health professionals who promoted PA and those who did not, considering their beliefs/attitudes, perceptions of capability, opportunity, and motivation (Table 1).

**Table 1:** Beliefs/attitudes and perceived capability, motivation, and opportunity among health professionals who promoted vs. did not promote PA in clinical practice

		Promotes physical activity						
		Yes		No		*U*	*p*	*d*
		*M*	*DP*	*M*	*DP*			
Believes that prescribing structured exercise is part of the health professional's role	Physicians	3.1	1.3	3.3	1.7	620.5	0.710	0.12
	Other health professionals	2.9	1.3	2.7	1.3	168.0	0.806	0.11
Considers that improving the services available for structured exercise for cancer patients is important	Physicians	4.4	0.7	4.6	0.7	680.5	0.333	0.27
	Other health professionals	4.6	0.6	4.4	0.8	156.0	0.528	0.34
Believes that encouraging structured exercise is relevant to improve patients’ health	Physicians	4.6	0.6	4.5	0.8	549.5	0.799	0.18
	Other health professionals	4.6	0.5	4.6	0.5	163.0	0.665	0.14
Believes that the presence of accredited exercise professionals in Health Services is important	Physicians	4.4	0.7	4.4	0.5	549.0	0.809	0.02
	Other health professionals	4.6	0.6	4.3	1.0	154.0	0.504	0.43
Believes that he/she would recommend accompanied structured exercise if reimbursed	Physicians	4.6	0.6	4.4	0.7	480.0	0.351	0.40
	Other health professionals	4.4	1.0	4.6	0.5	186.0	0.849	0.22
Considers having enough time in his/her clinical practice to recommend structured exercise	Physicians	2.2	1.2	2.1	1.0	563.0	0.915	0.10
	Other health professionals	3.0	1.3	2.1	1.1	114.0	0.118	0.64
Considers that the resources/services available for structured exercise for cancer patients are sufficient	Physicians	1.6	0.8	1.0	0.0	-*	-*	-*
	Other health professionals	1.9	1.0	1.6	0.5	148.5	0.446	0.37
Is motivated to promote structured exercise in his/her patients	Physicians	4.0	1.0	4.0	1.7	122.5	0.653	0.04
	Other health professionals	4.0	1.1	3.7	0.8	130.0	0.226	0.31
Likes to talk about exercise with his/her patients	Physicians	4.0	0.9	3.0	1.3	318.0	**0.025**	1.03
	Other health professionals	4.0	1.0	3.3	0.8	91.0	**0.027**	0.77
Encourages structured exercise because he/she believes it is good for patients’ health	Physicians	4.2	0.9	3.8	1.0	419.5	0.165	0.50
	Other health professionals	4.3	0.9	3.3	1.1	85	**0.017**	1.10
Knows the current exercise recommendations for cancer patients	Physicians	3.0	1.2	2.4	1.4	410.5	0.163	0.52
	Other health professionals	3.0	1.2	1.9	0.9	83.5	**0.020**	0.98
Feels that he/she has the skills to prescribe structured exercise	Physicians	2.0	1.1	1.6	0.7	462.0	0.323	0.40
	Other health professionals	2.3	1.4	1.3	0.5	109.0	*0.080*	0.74
Thinks that he/she has enough knowledge to answer specific questions about structured exercise	Physicians	2.3	1.0	2.0	1.1	457.0	0.308	0.35
	Other health professionals	2.5	1.2	1.6	0.8	99.0	*0.051*	0.79
Thinks he/she has the skills to monitor and supervise structured exercise	Physicians	1.7	0.9	1.6	0.5	596.5	0.856	0.10
	Other health professionals	2.1	1.3	1.4	0.8	125.5	0.179	0.54

-* variance is equal to 0, *M* mean, *SD* standard deviation, *U* Mann–Whitney test value, *p* significance level; *d*, Cohen’s *d*: 0.20–0.50, small effect size; 0.50–0.80, medium effect size; > 0.80, large effect size

As shown in Table 1, physicians who promoted PA were more likely to enjoy discussing PA with patients compared to those who did not (4.0 ± 0.9 vs. 3.0 ± 1.3, *p* = 0.025, *d* = 1.03). Among non-physician health professionals, those who promoted PA also reported greater enjoyment in discussing the topic (4.1 ± 1.0 vs. 3.3 ± 0.8, *p* = 0.027, *d* = 0.77), better knowledge of PA recommendations (3.0 ± 1.2 vs. 1.9 ± 0.9, *p* = 0.020, d* = *0.98), and more frequently encouraged PA due to its perceived health benefits (4.3 ± 0.9 vs. 3.3 ± 1.1, *p* = 0.017, *d* = 1.10). All these differences were of large magnitude.

As shown in Table 2, the differences between professionals who did and did not promote structured exercise were substantial and followed the same overall pattern for physicians and non-physicians. Professionals who reported promoting structured exercise also reported higher perceived capability, knowledge, and motivation to do so, together with stronger beliefs in the health benefits of exercise for their patients (*p* < 0.05; *d* = 0.54–0.85 among physicians and 0.62–1.19 among non-physicians). Physicians who promoted structured exercise would recommend it more often if reimbursed (4.8 ± 0.5 vs 4.5 ± 0.6, *p* < 0.001, *d* = 0.59), felt they had more consultation time to address PA (2.7 ± 1.3 vs 1.9 ± 0.9, *p* < 0.001, *d* = 0.74), and were more likely to consider that prescribing structured exercise was part of their medical role (3.3 ± 1.2 vs 2.9 ± 1.3, *p* = 0.040, *d* = 0.36); these differences were of small- to medium magnitude.

**Table 2:** Beliefs/attitudes and perceived capability, motivation, and opportunity among health professionals who promoted vs. did not promote structured exercise in clinical practice

		Promotes structured physical exercise						
		Yes		No		*U*	*p*	*d*
		*M*	*DP*	*M*	*DP*			
Believes that prescribing structured exercise is part of the health professional's role	Physicians	3.3	1.2	2.9	1.3	2061.5	**0.040**	0.36
	Other health professionals	3.1	1.3	2.6	1.3	247.5	0.151	0.41
Considers that improving the services available for structured exercise for cancer patients is important	Physicians	4.6	0.6	4.3	0.7	2216.0	*0.050*	0.36
	Other health professionals	4.7	0.5	4.6	0.6	288.5	0.448	0.20
Believes that encouraging structured exercise is relevant to improve patients’ health	Physicians	4.8	0.5	4.5	0.6	1895.0	**0.001**	0.54
	Other health professionals	4.8	0.4	4.5	0.6	234.0	**0.043**	0.61
Believes that the presence of accredited exercise professionals in Health Services is important	Physicians	4.4	0.8	4.4	0.7	2468.0	0.685	0.03
	Other health professionals	4.6	0.6	4.5	0.7	281.5	0.373	0.27
Believes that he/she would recommend accompanied structured exercise if reimbursed	Physicians	4.8	0.5	4.5	0.6	1825.5	** < 0.001**	0.59
	Other health professionals	4.6	0.6	4.1	1.2	245.0	0.100	0.56
Considers having enough time in his/her clinical practice to recommend structured exercise	Physicians	2.7	1.3	1.9	0.9	1643.0	** < 0.001**	0.74
	Other health professionals	3.3	1.2	2.6	1.4	230.0	*0.076*	0.51
Considers that the resources/services available for structured exercise for cancer patients are sufficient	Physicians	1.7	0.9	1.6	0.6	652.0	0.637	0.03
	Other health professionals	2.1	1.7	1.7	0.6	270.0	0.295	0.43
Is motivated to promote structured exercise in his/her patients	Physicians	4.2	0.9	3.7	1.0	424.0	**0.019**	0.54
	Other health professionals	4.4	0.9	3.6	1.2	190.5	**0.008**	0.76
Likes to talk about exercise with his/her patients	Physicians	4.3	0.9	3.7	0.9	1519.0	** < 0.001**	0.72
	Other health professionals	4.4	0.7	3.6	1.0	161.5	**0.001**	0.94
Encourages structured exercise because he/she believes it is good for patients’ health	Physicians	4.6	0.7	3.9	1.0	1500.5	** < 0.001**	0.77
	Other health professionals	4.7	0.5	3.8	1.0	142.0	** < 0.001**	1.19
Knows the current exercise recommendations for cancer patients	Physicians	3.4	1.3	2.7	1.1	1697.5	** < 0.001**	0.62
	Other health professionals	3.3	1.2	2.6	1.1	218.5	**0.045**	0.62
Perceives that he/she has the skills to prescribe structured exercise	Physicians	2.4	1.2	1.7	0.9	1695.0	** < 0.001**	0.68
	Other health professionals	2.8	1.5	1.6	0.8	182.5	**0*.*006**	0.93
Thinks that he/she has enough knowledge to answer specific questions about structured exercise	Physicians	2.8	1.0	2.0	0.8	1464.0	** < 0.001**	0.85
	Other health professionals	2.8	1.3	2.1	1.0	229.5	*0.072*	0.56
Thinks he/she has the skills to monitor and supervise structured exercise	Physicians	2.0	1.1	1.5	0.6	1871.0	**0.002**	0.62
	Other health professionals	2.6	1.5	1.5	0.7	194.5	**0.011**	0.89

*M* mean, *SD* standard deviation, *U* Mann–Whitney test value, *p* significance level; *d*, Cohen’s *d*: 0.20–0.50, small effect size; 0.50–0.80, medium effect size; > 0.80, large effect size

Table 3 summarizes the associations between beliefs/attitudes, COM-B perceptions and promotion practices. Among physicians, the proportion of consultations in which PA was promoted increased with greater perceived time/opportunity (rho = 0.35, *p* < 0.001), motivation (rho = 0.30, *p* = 0.011), enjoyment in discussing the topic (rho = 0.32, *p* < 0.001), and belief in its patient benefits (rho = 0.23, *p* = 0.005). Higher perceived competence and knowledge were likewise linked to promoting activity in more consultations (rho = 0.27–0.32, *p* < 0.001). A similar pattern was observed for (i) consultation time specifically devoted to activity counseling and (ii) referrals to exercise physiologists—both behaviors increased with greater perceived opportunity, motivation and competence (rho’s = 0.18–0.35, *p* < 0.05). Viewing structured exercise prescription as part of the physician’s remit showed a weaker but significant association with time spent on activity counseling and with referral habits (rho = 0.18, *p* < 0.05).

**Table 3:** Correlations between health professionals' beliefs/attitudes, perceived capability, opportunity, and motivation and their PA promotion practices

	Physicians			Other health professionals	
	% of consultations promoting physical activity	Consultation time dedicated to promoting physical activity	Referral habit to exercise physiologists	% patients recommend Physical Activity	% of patients recommend Physical Exercise
Believes that prescribing structured exercise is part of the health professional's role	0.13	0.18*	0.18*	0.02	0.04
Considers that improving the services available for structured exercise for cancer patients is important	0.13	0.13	0.11	−0.02	−0.17
Believes that encouraging structured exercise is relevant to improve patients' health	0.06	0.11	0.17*	0.11	0.06
Believes that the presence of accredited exercise professionals in Health Services is important	0.10	0.03	0.11	0.08	0.03
Believes that he/she would recommend accompanied structured exercise if reimbursed	0.11	0.03	0.08	0.03	0.02
Considers having enough time in his/her clinical practice to recommend structured exercise	0.35***	0.34***	0.27***	0.37**	0.17
Considers that the resources/services available for structured exercise for cancer patients are sufficient	0.03	0.17	0.20#	0.06	0.12
Is motivated to promote structured exercise in his/her patients	0.30*	0.31*	0.27*	0.15	0.16
Likes to talk about exercise with his/her patients	0.32***	0.14	0.23**	0.29*	0.35*
Encourages structured exercise because he/she believes it is good for patients' health	0.23**	0.20*	0.24**	0.24#	0.26#
Knows the current exercise recommendations for cancer patients	0.32***	0.18*	0.35***	0.49***	0.53***
Perceives that he/she has the skills to prescribe structured exercise	0.29***	0.23**	0.25**	0.19	0.33*
Thinks that he/she has enough knowledge to answer specific questions about structured exercise	0.28***	0.34***	0.32***	0.08	0.20
Thinks he/she has the skills to monitor and supervise structured exercise	0.27***	0.24**	0.21*	0.02	0.22

Cells show Spearman’s correlation coefficients (rho)

*p < 0.05; **p < 0.01; ***p < 0.001

For non-physician professionals, medium-magnitude positive associations emerged between the proportion of patients advised to be active and (i) perceived time/opportunity to promote activity (rho = 0.37, *p* = 0.007), (ii) knowledge of activity guidelines (rho = 0.49, *p* < 0.001), and (iii) enjoyment in discussing activity (rho = 0.29, *p* = 0.040). The percentage of patients receiving structured exercise recommendations increased with greater guideline knowledge (rho = 0.53, *p* < 0.001), competence to prescribe exercise (rho = 0.33, *p* = 0.017), and enjoyment of the topic (rho = 0.35, *p* = 0.011); these associations were medium to strong in magnitude.

Based on the beliefs/attitudes and COM-B variables that showed significant associations with PA and exercise promotion practices, exploratory regression analyses were conducted.

Among physicians, the logistic regression model predicting structured exercise promotion (*χ*^2^ = 8.252, *p* = 0.004, *R*^2^ = 0.29) identified two main predictors: the belief that they would recommend structured exercise if reimbursed (OR = 4.68, *p* = 0.007) and the perceived availability of time in consultations (OR = 2.81, *p* < 0.001).

Regarding the percentage of consultations in which PA was promoted (*F* = 16.367, *p* < 0.001, *R*^2^ = 0.19), only perceived opportunity/time remained a significant predictor (*β* = 0.44, *p* < 0.001), suggesting that having enough time was a key facilitator of PA promotion.

Considering time spent during consultations specifically promoting PA (*F* = 18.695, *p* = 0.003, *R*^2^ = 0.46), the most relevant predictors were as follows: perceived competence to prescribe exercise (*β* = 0.50, *p* < 0.001, *R*^2^-change = 0.32); perceived opportunity/time (*β* = 0.44, *p* < 0.001, *R*^2^-change = 0.05); and belief that prescribing exercise was part of the physician’s role, which had a negative association (*β* = −0.32, *p* = 0.003, *R*^2^-change = 0.08). For the habit of referring patients to exercise physiologists (*F* = 7.169, *p* < 0.001, *R*^2^ = 0.25), the most influential variables were as follows: perceived opportunity/time (*β* = 0.41, *p* = 0.003, *R*^2^-change = 0.14), belief that prescribing structured exercise is a medical responsibility (*β* = 0.27, *p* = 0.025, *R*^2^-change = 0.06), and perceived ability to monitor/supervise structured exercise, which was negatively associated (*β* = −0.28, *p* = 0.034, *R*^2^ change = 0.05).

Among non-physician healthcare professionals, perceived knowledge of exercise recommendations for cancer patients emerged as the most relevant predictor of PA promotion. The logistic regression model predicting this behavior (*χ*^*2*^ = 5.914, *p* = 0.015, *R*^2^ = 0.10) showed that professionals who felt more familiar with the guidelines were significantly more likely to promote PA (OR = 2.49, *p* = 0.031).

In contrast, when predicting the promotion of structured exercise, the regression model (*χ*^*2*^ = 5.755, *p* = 0.016, *R*^2^ = 0.37) revealed that professionals who reported stronger motivation to promote PA due to its health benefits (OR = 6.31, *p* = 0.005) and those who felt more competent to prescribe structured exercise (OR = 2.00, *p* = 0.032) were significantly more likely to engage in this practice.

Among non-physician professionals, two variables accounted for most of the variance in how many patients received general PA advice (*F* = 10.435, *p* < 0.001, *R*^2^ = 0.30). First, practitioners who felt well-informed about exercise guidelines for oncology patients counseled a larger proportion of patients (*β* = 0.35, *p* = 0.007; *R*^2^-change = 0.20). Second, a greater enjoyment in discussing PA with patients was also a significant contributor (*β* = 0.33, *p* = 0.012; *R*^2^-change = 0.10). When the outcome was structured exercise counseling, knowledge of oncology-specific exercise recommendations was the only significant predictor (*F* = 20.427, *p* < 0.001, *R*^2^ = 0.29; *β* = 0.54, *p* < 0.001).

## Discussion

This study aimed to examine healthcare professionals’ opinions and practices regarding PA and structured exercise in oncology care, and to analyze whether their beliefs/attitudes and their perceptions of capability, opportunity, and motivation were associated with their actual promotion behaviors.

Overall, results showed that among physicians, PA and exercise promotion practices were particularly associated with their perceived opportunity/time during consultations to address these behaviors. In addition, higher knowledge of exercise guidelines for cancer patients, stronger perceived competence to prescribe structured exercise, and better-quality motivation—reflected in enjoyment in discussing the topic and valuing the health benefits of exercise—were also positively linked with PA promotion behaviors. These patterns echo the findings of an international scoping survey of 375 oncology practitioners, which reported that 35–50% of respondents had poor knowledge of when, how, and whom to refer to exercise programs, and that guideline recall independently predicted counseling and referral behavior [18]. The same survey ranked limited time, safety concerns, and unclear referral pathways among the top barriers, mirroring the prominence of perceived opportunity in our physician sample. Certain beliefs also played a role: physicians who believed that prescribing structured exercise was part of their professional role, or who stated they would be more likely to recommend exercise if it were reimbursed, were more prone to promote it in practice. In turn, among non-medical health professionals, motivational and capability-related factors seemed to play a more prominent role. Specifically, greater knowledge of exercise guidelines for oncology, stronger perceived competence to prescribe structured exercise, greater enjoyment in discussing PA, and stronger beliefs in its benefits for patients were the variables most closely linked to higher levels of PA promotion. These findings suggest that among non-medical professionals, higher quality motivation and perceived competence are central to engaging in exercise promotion practices.

The most prominent factor associated with physicians’ promotion of PA was their enjoyment in discussing exercise with patients, which reflects intrinsic motivation to promote PA. In contrast, the key determinant of structured exercise promotion was physicians’ perceived opportunity—specifically, the feeling of having sufficient time during consultations to recommend exercise. Additionally, the perceived competence to prescribe structured exercise emerged as a critical predictor of the amount of time physicians dedicated to promoting PA during consultations. On the other hand, among non-medical health professionals, the most relevant factors included perceived knowledge and perceived competence to prescribe structured exercise for oncology patients. Moreover, enjoyment in discussing PA and a strong belief in its health benefits were also significantly associated with higher levels of promotion. These findings align closely with the COM-B model [16], which identifies motivation, opportunity, and perceived capability as the key determinants of behavior—in this case, the promotion of PA and structured exercise in oncology care. Furthermore, they are consistent with Self-Determination Theory, which posits that greater autonomous motivation (i.e., engaging in a behavior out of enjoyment or personal value) and the satisfaction of the basic psychological need for competence (feeling capable of performing a task effectively) are associated with more sustained and committed engagement in a behavior [19]. Our results also corroborate prior evidence showing that greater knowledge of PA guidelines is linked to increased promotion practices, whereas low perceived competence acts as a barrier to such promotion [9–12]. In this regard, our hypothesis suggesting that lower perceptions of competence, knowledge, opportunity, and motivation would be associated with less frequent promotion of PA among oncologists and other health professionals was confirmed in both groups. Importantly, this study further emphasizes that the relative weight of these factors differs between medical and non-medical professionals, highlighting the need for tailored strategies when designing interventions to support the promotion of PA across multidisciplinary oncology teams.

Conversely, the hypothesis proposing that more favorable beliefs among oncology professionals would enhance their promotion practices of PA in cancer patients was also confirmed in both groups. Physicians who believed that prescribing structured exercise was part of their role, who recognized its relevance for patient health, and who were convinced that they would recommend more exercise if it were subsidized, reported higher promotion of structured exercise and were more likely to refer patients to exercise physiologists. Regarding non-medical health professionals, beliefs generally played a less prominent role in explaining their promotion practices. However, those who enjoyed discussing exercise with patients and believed in its benefits were more likely to promote structured exercise. These findings reinforce previous evidence demonstrating that positive beliefs and attitudes toward PA in cancer patients directly influence and enhance physicians' promotion practices [13, 16, 20–23], suggesting that this influence, at least partially, extends to non-medical health professionals as well.

Interestingly, the belief that prescribing structured exercise is part of the physicians’ role was identified as one of the most explanatory factors for the promotion of structured exercise among physicians; however, its effect unexpectedly reversed, becoming a negative predictor. This finding contradicts the positive trend observed in the other variables and may be explained by the correlation and interaction between variables included in the regression model. It is possible that other more influential factors, namely, perceived competence to prescribe structured exercise and perceived opportunity/time to promote PA during consultations, suppressed the effect of this belief and even reversed its regression coefficient.

### Strengths and limitations

This study has several limitations. First, its cross-sectional observational design precludes causal inference between independent and dependent variables. Second, the samples were not fully representative, as the final numbers fell short of the a-priori targets, limiting generalizability. The non-physician subgroup was particularly small, which may have reduced power to detect some associations or group differences. Third, recruitment was convenience-based: although the survey link was disseminated through multiple channels (professional associations, e-mail lists, conferences, academic networks), the response rate could not be calculated, and selection bias is likely—clinicians who value PA may have been more inclined to participate. Fourth, data were collected between 2019 and 2024, a period encompassing the COVID-19 pandemic: heightened workloads and shifting priorities during that time may have influenced both promotion practices and self-reported perceptions. Finally, social desirability effects cannot be discarded, as it is possible that health professionals have overestimated their PA promotion practices to correspond to social expectations.

Despite these limitations, this study also has important strengths. Notably, it includes an exclusive sample of non-medical oncology professionals, whereas most previous research has focused on oncologists alone or on mixed samples without stratified analysis. By analyzing physicians and non-physicians separately, the study provides a broader—and crucially multidisciplinary—view of beliefs/attitudes and COM-B constructs (capability, opportunity, motivation) in relation to PA promotion. Finally, the work is situated in the Portuguese healthcare context, adding much-needed evidence from a setting that has been under-represented in the literature.

### Practical implications

The findings of this study carry relevant implications for the clinical practice of healthcare professionals involved in oncology care, including both physicians and non-physicians. Although many professionals acknowledge the importance of PA, only about half report promoting structured exercise, which may be due to gaps in knowledge and perceived competence regarding exercise prescription. These findings highlight the need to reinforce professional training—particularly by integrating content on PA guidelines for cancer patients into medical curricula. This should include information on safety, clinical precautions, and red flags during exercise prescription and supervision. This might also contribute to reducing physicians’ perceived lack of time to address the topic, as it will help them prioritize information and organize it in a more concise way.

Such educational efforts aim not only to enhance knowledge and skills but also to increase the perceived value of exercise in cancer care and highlight the role of qualified exercise professionals within health services. This reinforces the need for interdisciplinary collaboration in oncology settings. Therefore, ongoing professional development should be prioritized, with a dual focus on technical proficiency and the self-confidence and perceived competence necessary to address PA with patients in an informed and effective manner.

## Conclusion

This study examined how oncology professionals’ beliefs/attitudes and their perceived capability, opportunity, and motivation related to the promotion of PA and structured exercise. Among physicians, the strongest drivers were (i) enjoyment of talking about exercise (intrinsic motivation), (ii) perceived opportunity—having enough time in the consultation, and (iii) perceived competence to prescribe structured exercise. For non-medical staff, the leading predictors were knowledge of PA guidelines, confidence to prescribe, enjoyment of the topic, and valuation of its benefits.

Future research should confirm these patterns in larger samples and using longitudinal designs to establish cause-and-effect relationships. Equally important is the development and testing of targeted educational interventions that go beyond boosting knowledge and self-efficacy to demonstrate measurable gains in day-to-day promotion practices. Finally, a deeper exploration of barriers and facilitators across specialties and clinical contexts will be essential for designing implementation strategies that make exercise promotion a routine part of multidisciplinary oncology care. 

## Data Availability

No datasets were generated or analysed during the current study.

## Declarations

### Data confidentiality

All data were anonymized and handled confidentially, ensuring participants’ privacy.
